# Diet and risk for acute tubulointerstitial nephritis

**DOI:** 10.1097/MD.0000000000038443

**Published:** 2024-06-28

**Authors:** Yanjiang Yang, Wenwen Yang

**Affiliations:** a Department of Rheumatology and Immunology, The People’s Hospital of Qiandongnan Autonomous Prefecture, Kaili, Guizhou Province, China; b The First Clinical Medical College, Lanzhou University, Lanzhou, Gansu Province, China.

**Keywords:** acute tubulointerstitial nephritis, dietary intake, FinnGen biobank, Mendelian randomization, UK Biobank

## Abstract

Uncertainty exists regarding the association between diet and acute tubulointerstitial nephritis. Dietary factors served as exposures, including intake of alcohol, beef, non-oily fish, fresh fruit, oily fish, dried fruit, coffee, salad/raw vegetable, cereal, tea, water, salt, cooked vegetable, cheese, poultry, pork, Lamb/mutton, bread, and processed meat were extracted from the UK Biobank. Acute tubulointerstitial nephritis served as the outcome extracted from the FinnGen biobank. The 3 main methods of this analysis were weighted median, inverse-variance-weighted (IVW), and MR-Egger methods. The heterogeneity was measured employing Cochran Q test. The MR-PRESSO method was employed to identify possible outliers. The robustness of the IVW method was evaluated by employing the leave-one-out analysis. According to the IVW method, processed meat intake (OR = 0.485; *P* = .00152), non-oily fish intake (OR = 0.396; *P* = .0454), oily fish intake (OR = 0.612; *P* = .00161), and dried fruit intake (OR = 0.536; *P* = .00648) reduced the risk of acute tubulointerstitial nephritis. Other dietary factors were not shown to be causally related to acute tubulointerstitial nephritis. This study revealed that intake of processed meat, non-oily fish, oily fish, and dried fruit all decreased the risk of acute tubulointerstitial nephritis.

## 1. Introduction

Councilman^[1]^ published the first description of acute tubulointerstitial nephritis in 1898. As an acute inflammatory disease, acute tubulointerstitial nephritis involves the renal interstitium and tubules but rarely affects the glomeruli and blood vessels.^[1]^ Acute tubulointerstitial nephritis has a variety of etiologies, including idiopathic, immune-mediated, infectious, systemic disease, and drugs (including antibiotics and NSAIDs).^[2]^ Diet is a crucial non-pharmaceutical intervention in the prevention and treatment of diseases.^[3–6]^ The impact of diet on acute tubulointerstitial nephritis, however, has been little studied. Using Mendelian randomization (MR) analysis methods, we explored the associations between dietary variables and acute tubulointerstitial nephritis and discovered that intake of processed meat, non-oily fish, oily fish, and dried fruit decreased the incidence of acute tubulointerstitial nephritis.

## 2. Methods

MR is a method of using genetic variations, Mainly single-nucleotide polymorphisms (SNPs), as instrumental variables (IVs) to identify the causal effect of exposures on outcomes.^[7]^ MR provides an alternative method for identifying the causality in epidemiological studies. MR requires the fulfillment of 3 basic assumptions.^[8]^ First, the IVs must be strongly correlated with exposure factors. Second, the IVs had no relationship to any conceivable confounding variables. Third, the IVs and outcomes cannot be associated directly. The study was waived from the approval of the institutional review board because it employed the de-identified and publicly available data from the University of Bristol MRC Integrative Epidemiology Unit (IEU) Open GWAS project.

### 2.1. Data sources and the choice of IVs

Dietary factors served as exposures in this study included intake of alcohol, beef, non-oily fish, fresh fruit, oily fish, dried fruit, coffee, salad/raw vegetable, cereal, tea, water, salt, cooked vegetable, cheese, poultry, pork, Lamb/mutton, bread, and processed meat. Hundreds of thousands of European participants are involved in each of the dietary factors mentioned above. The IEU open GWAS project, funded by the University of Bristol MRC IEU, directly or indirectly extracted these GWAS data from the UK Biobank. The GWAS data of acute tubulointerstitial nephritis (including acute pyelitis, acute infectious interstitial nephritis, and acute pyelonephritis^[9]^) was taken from the FinnGen biobank. Acute tubulointerstitial nephritis as the outcome in this study included 11,216 European-descent cases and 201,028 European-descent controls. Table 1 and Supplementary Table 1, http://links.lww.com/MD/N40 provide more details regarding the outcome and exposure datasets. The IVs used in our study were selected employing the following conditions. First, the genome-wide significance threshold was *P* < 5 × 10^−8^, the clumping window was 10,000 kb, and the level of linkage disequilibrium r2 < 0.001. Second, the IVs’ F statistics need to be in excess of 10. Third, palindromic and missing SNPs will be removed directly.

**Table 1:** Information on exposure and outcome datasets.

IEU GWAS id	Exposure or outcome	Identified SNPs	Participants included in analysis	F-statistic
ieu-b-73	Alcoholic drinks per week	33	335394 European-descent individuals	98.599
ukb-b-5779	Alcohol intake frequency	92	462346 European-descent individuals	115.197
ukb-b-6324	Processed meat intake	23	461981 European-descent individuals	39.547
ukb-b-8006	Poultry intake	7	461900 European-descent individuals	24.411
ukb-b-2862	Beef intake	14	461053 European-descent individuals	27.841
ukb-b-17627	Non-oily fish intake	11	460880 European-descent individuals	27.544
ukb-b-2209	Oily fish intake	60	460443 European-descent individuals	38.088
ukb-b-5640	Pork intake	13	460162 European-descent individuals	18.761
ukb-b-14179	Lamb/mutton intake	30	460006 European-descent individuals	19.720
ukb-b-11348	Bread intake	25	452236 European-descent individuals	38.339
ukb-b-1489	Cheese intake	60	451486 European-descent individuals	44.880
ukb-b-8089	Cooked vegetable intake	17	448651 European-descent individuals	20.818
ukb-b-6066	Tea intake	39	447485 European-descent individuals	62.782
ukb-b-3881	Fresh fruit intake	52	446462 European-descent individuals	15.502
ukb-b-15926	Cereal intake	38	441640 European-descent individuals	32.753
ukb-b-1996	Salad/ raw vegetable intake	18	435435 European-descent individuals	17.571
ukb-b-5237	Coffee intake	38	428860 European-descent individuals	41.751
ukb-b-16576	Dried fruit intake	39	421764 European-descent individuals	25.209
ukb-b-8121	Salt added to food	96	462630 European-descent individuals	36.232
ukb-b-14898	Water intake	37	427588 European-descent individuals	35.982
finn-b-N14_PYELONEPHR	Acute tubulointerstitial nephritis	NA	11216 European-descent cases and 201028 European-descent controls	NA

The information of the exposure and outcome datasets.

GWAS = genome-wide association studies, IEU = integrative epidemiology unit, SNPs = single-nucleotide polymorphisms.

### 2.2. Statistical analysis

This analysis was concluded using Three MR methods. Firstly, the primary method for identifying causality: the inverse-variance-weighted (IVW) method. The strongest capacity to identify causality is provided by the IVW method, which demands that horizontal pleiotropy is balanced or all SNPs are valid.^[10]^ Secondly, the complementary method for identifying causality: the MR–Egger and Weighted Median method. If their results confirm the IVW method, the conclusions’ reliability will be greatly increased. The horizontal pleiotropy can be detected by employing the MR-Egger method, which allows the occurrence of non-zero intercepts. The heterogeneity was measured employing Cochran Q test. The MR-PRESSO method was employed to identify possible outliers. The robustness of the IVW method was evaluated by employing the leave-one-out analysis. All analyses were completed utilizing the TwoSampleMR package^[11]^ and R program (version 4.2.0).

## 3. Results

As can be observed in Table 1, the F-statistic for IVs is >10, demonstrating that strong associations existed between IVs and exposures. There were no outliers detected by the MR-PRESSO method. The horizontal pleiotropy was not found using the MR-Egger method. Our results primarily presented the analysis findings of the IVW method, as it possessed the most sensitive ability to detect causal relationships.

**Figure 1.**
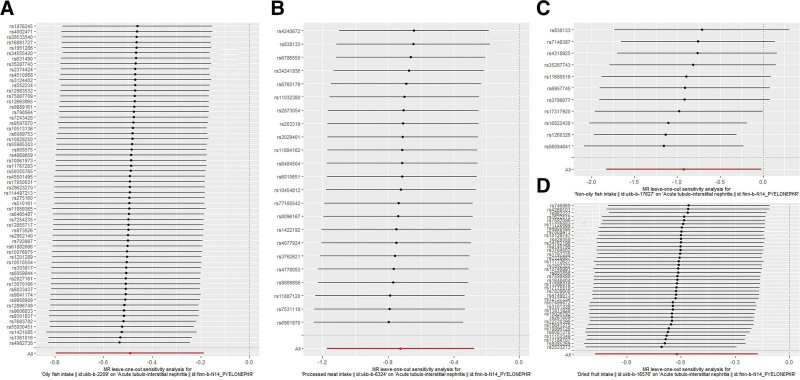
The results of leave-one-out analyses. (A) Oily fish intake, (B) processed meat intake, (c) non-oily fish intake, and (d) dried fruit intake.

Ultimately, 4 dietary factors were identified to be associated with acute tubulointerstitial nephritis. According to the IVW method, processed meat intake (OR = 0.485; *P* = .00152), non-oily fish intake (OR = 0.396; *P* = .0454), oily fish intake (OR = 0.612; *P* = .00161), and dried fruit intake (OR = 0.536; *P* = .00648) reduced the risk of acute tubulointerstitial nephritis. Other dietary factors were not shown to be causally related to acute tubulointerstitial nephritis. According to the weighted median method, processed meat intake (OR = 0.396; *P* = .00334), non-oily fish intake (OR = 0.299; *P* = .0339), oily fish intake (OR = 0.602; *P* = .0207), salad/raw vegetable intake (OR = 0.278; *P* = .0254) and dried fruit intake (OR = 0.452; *P* = .00987) reduced the risk of acute tubulointerstitial nephritis. Only oily fish intake (OR = 0.197; *P* = .0143) was observed to decrease the risk of acute tubulointerstitial nephritis in the MR-Egger method. Table 2 provides more analytical outcomes. The leave-one-out analysis showed that the causal relationships between processed meat intake, oily fish intake, and dried fruit intake and acute tubulointerstitial nephritis were not affected by a single SNP. Figure 1 exhibits the results of the leave-one-out analysis.

**Table 2:** The results of Mendelian randomization analyses.

	Exposure	Used SNPs	Inverse variance-weighted method		Weighted median method		MR-Egger method		Cochrane Q test		Pleiotropy			MR-PRESSO						
			OR(95% CI)	*P* value	OR(95% CI)	*P* value	OR(95% CI)	*P* value	Q	*P* value	MR-Egger intercept	se	*P* value	Raw			Outliers	Outlier-corrected		
														Casual estimate	sd	*P* value		Casual estimate	sd	*P* value
ieu-b-73	Alcoholic drinks per week	33	1.125 (0.798–1.586)	.502	1.270 (0.769–2.098)	.350	1.862 (0.852–4.070)	.129	32.062	.464	−0.00956	0.00681	.170	0.118	0.175	.506	NA	NA	NA	NA
ukb-b-5779	Alcohol intake frequency	92	1.069 (0.924–1.236)	.369	1.129 (0.911–1.398)	.268	0.829 (0.530–1.297)	.413	98.159	.286	0.00639	0.00542	.242	0.0665	0.0741	.372	NA	NA	NA	NA
ukb-b-6324	Processed meat intake	23	0.485 (0.310–0.758)	.00152	0.396 (0.213–0.735)	.00334	0.147 (0.0156–1.389)	.109	17.147	.755	0.0181	0.0170	.300	−0.724	0.202	.00162	NA	NA	NA	NA
ukb-b-8006	Poultry intake	7	1.515 (0.538–4.270)	.432	1.396 (0.346–5.635)	.6390	4.840e-05 (1.496e-18 - 1.565e + 09)	.559	3.728	.713	0.112	0.172	.543	0.416	0.417	.357	NA	NA	NA	NA
ukb-b-2862	Beef intake	14	1.392 (0.705–2.748)	.340	1.485 (0.581–3.797)	.409	14.092 (0.234–847.091)	.230	7.651	.866	−0.0294	0.0262	.283	0.331	0.266	.236	NA	NA	NA	NA
ukb-b-17627	Non-oily fish intake	11	0.396 (0.160–0.981)	.0454	0.299 (0.0980–0.913)	.0339	0.0249 (0.000382–1.623)	.117	13.445	.200	0.0343	0.0259	.217	−0.927	0.463	.0733	NA	NA	NA	NA
ukb-b-2209	Oily fish intake	60	0.612 (0.451–0.830)	.00161	0.602 (0.392–0.925)	.0207	0.197 (0.0559–0.696)	.0143	68.119	.195	0.0169	0.00930	.0752	−0.492	0.156	.00253	NA	NA	NA	NA
ukb-b-5640	Pork intake	13	1.115 (0.467–2.663)	.807	1.030 (0.300–3.536)	.962	1.967 (0.00679–569.181)	.819	13.107	.361	−0.00589	0.0296	.846	0.109	0.444	.811	NA	NA	NA	NA
ukb-b-14179	Lamb/mutton intake	30	0.855 (0.490–1.492)	.582	0.949 (0.445–2.026)	.892	0.153 (0.0147–1.598)	.128	21.870	.826	0.0191	0.0129	.150	−0.156	0.246	.531	NA	NA	NA	NA
ukb-b-11348	Bread intake	25	0.765 (0.498–1.176)	.222	0.743 (0.394–1.400)	.358	2.462 (0.337–17.990)	.384	22.822	.530	−0.0170	0.0144	.250	−0.268	0.214	.222	NA	NA	NA	NA
ukb-b-1489	Cheese intake	60	0.793 (0.602–1.045)	.0993	0.679 (0.456–1.011)	.0566	1.633 (0.508–5.251)	.414	69.214	.171	−0.0125	0.0100	.217	−0.232	0.141	.10500	NA	NA	NA	NA
ukb-b-8089	Cooked vegetable intake	17	1.236 (0.421–3.633)	.700	1.879 (0.590–5.984)	.286	0.905 (4.452e-06 - 1.840e + 05)	.987	37.487	.00179	0.00322	0.0641	.961	0.212	0.550	.705	NA	NA	NA	NA
ukb-b-6066	Tea intake	39	0.982 (0.747–1.290)	.895	0.747 (0.485–1.150)	.185	0.719 (0.397–1.303)	.284	39.782	.391	0.00668	0.00579	.256	−0.0183	0.139	.896	NA	NA	NA	NA
ukb-b-3881	Fresh fruit intake	52	0.803 (0.494–1.305)	.376	0.548 (0.267–1.124)	.101	0.271 (0.0527–1.391)	.124	55.640	.304	0.01050	0.00768	.179	−0.219	0.248	.380	NA	NA	NA	NA
ukb-b-15926	Cereal intake	38	1.091 (0.758–1.570)	.641	1.221 (0.725–2.055)	.453	0.986 (0.208–4.672)	.986	26.675	.895	0.00147	0.0113	.897	0.0867	0.158	.586	NA	NA	NA	NA
ukb-b-1996	Salad/ raw vegetable intake	18	0.391 (0.143–1.069)	.0672	0.278 (0.0907–0.855)	.0254	0.0168 (0.000183–1.541)	.095	26.838	.0605	0.0341	0.0244	.181	−0.939	0.513	.0848	NA	NA	NA	NA
ukb-b-5237	Coffee intake	38	0.873 (0.632–1.207)	.412	0.799 (0.487–1.311)	.375	0.974 (0.508–1.869)	.938	36.115	.510	−0.00207	0.00545	.706	−0.136	0.163	.411	NA	NA	NA	NA
ukb-b-16576	Dried fruit intake	39	0.536 (0.342–0.840)	.00648	0.452 (0.247–0.826)	.00987	0.511 (0.0679–3.844)	.518	48.303	.122	0.000592	0.0125	.963	−0.624	0.229	.00973	NA	NA	NA	NA
ukb-b-8121	Salt added to food	96	1.042 (0.830–1.309)	.722	1.004 (0.719–1.404)	.979	0.707 (0.329–1.521)	.377	92.203	.562	0.00574	0.00552	.301	0.0414	0.115	.719	NA	NA	NA	NA
ukb-b-14898	Water intake	37	0.815 (0.572–1.162)	.259	1.053 (0.629–1.763)	.845	0.939 (0.339–2.601)	.905	35.486	.493	−0.00225	0.00774	.773	−0.204	0.180	.263	NA	NA	NA	NA

CI = confidence interval, NA = not available, OR = odds ratio, SNPs = single-nucleotide polymorphisms.

## 4. Discussion

Acute kidney failure can result from tubulointerstitial nephritis, which is characterized by infiltration of inflammatory cells in the renal interstitium.^[12]^ Acute tubulointerstitial nephritis has a wide range of causes, including medications, infections, Immune-Mediated, and other factors.^[13]^ However, the effect of dietary factors in the development of acute tubulointerstitial nephritis has been disregarded. This is understandable given that there are very few studies on how nutrition affects nephritis, making it challenging to identify an association between diet and nephritis, particularly acute nephritis. The dietary factor is a difficult indicator to measure, and one study^[14]^ comparing a low-protein diet to a typical diet to assess if diet had an impact on acute nephritis failed to discover a positive result, possibly as a result of the study limitations involving the inclusion of only 57 participants and the study brief follow-up time (no less than 1 year). Using randomized controlled methods to investigate the impact of dietary factors on tubulointerstitial nephritis is very challenging since dietary habits are challenging to alter. Observational studies require a large number of participants to be sufficiently convincing, and the level of evidence for observational studies is relatively low. This Two-sample MR analysis was thus performed. MR studies have some unique benefits. First, MR studies were able to include a larger number of participants. Secondly, reverse causality and confounding factors can be efficiently avoided in MR studies.^[15]^ Using the MR analysis method, this study found that processed meat intake, non-oily fish intake, oily fish intake, and dried fruit intake reduced the risk of acute tubulointerstitial nephritis. Other dietary factors included in this study were not associated with acute tubulointerstitial nephritis. It is unclear how dietary factors influence acute tubule-interstitial nephritis. We were unable to discover any studies that addressed how dietary factors may influence acute tubule-interstitial nephritis.

This MR study revealed correlations between intake of processed meat, non-oily fish, oily fish, and dried fruit and acute tubulointerstitial nephritis, and we must pay special attention to interpreting these results. First, the causality identified by the MR analysis represented the results of repeated exposure to dietary factors. Exposure for short periods may not have a clinical impact as a result. Second, only the overall impacts of exposures on outcomes, not the direct impacts, were revealed by MR analyses. Exposures and outcomes may be connected by incredibly intricate mechanisms.

This study inevitably includes a few limitations. First, we were unable to identify whether there was a U-shaped relationship (e.g., the risk of developing tubulointerstitial nephritis increases and then decreases as certain dietary factors increase.) between dietary factors and acute tubule-interstitial nephritis considering we used continuous data on dietary factors. Second, we were unable to perform stratified analyses by sex and age owing to the absence of GWAS data for these 2 demographics. Third, we are unable to further divide different dietary intake types, which restricts more thorough analysis. Fourth, extrapolating our findings to other populations is challenging for the reason our analysis mainly focuses on populations from Europe.

## 5. Conclusion

This study demonstrated a correlation between dietary factors and acute tubulointerstitial nephritis. This study revealed that intake of processed meat, non-oily fish, oily fish, and dried fruit all decreased the risk of acute tubulointerstitial nephritis. While other dietary factors are unrelated to acute tubulointerstitial nephritis.

## Acknowledgments

Special thanks to the IEU open GWAS project developed by The MRC Integrative Epidemiology Unit (IEU) at the University of Bristol. Thank them for extracting relevant GWAS summary-level data from published articles, UK Biobank, and FinnGen Biobank.

## Author contributions

**Conceptualization:** Yanjiang Yang, Wenwen Yang.

**Data curation:** Yanjiang Yang.

**Formal analysis:** Yanjiang Yang.

**Funding acquisition:** Yanjiang Yang.

**Investigation:** Yanjiang Yang.

**Methodology:** Yanjiang Yang.

**Project administration:** Yanjiang Yang.

**Resources:** Wenwen Yang.

**Software:** Wenwen Yang.

**Validation:** Yanjiang Yang, Wenwen Yang.

**Visualization:** Yanjiang Yang.

**Writing – original draft:** Yanjiang Yang.

## Supplementary Material


