# Supplementary material for: Diet and risk for acute tubulointerstitial nephritis
Source: Medicine (Baltimore). 2024 Jun 28;103(26):e38443. doi: 10.1097/MD.0000000000038443 (PMC11466169; doi:10.1097/MD.0000000000038443)
Supplement: Supplementary file 1 [file medi-103-e38443-s001.docx]

Supplementary Table 1

| Exposure or outcome | definition | Source/Detailed Description/Results |
| --- | --- | --- |
| Alcoholic drinks per week | 1. Defined as the average number of drinks a participant reported drinking each week, aggregated  across all types of alcohol. If a study recorded binned response ranges (e.g., 1-4 drinks per week,  5-10 drinks per week) we used the midpoint of the range. For example, if an individual reported 1-5  drinks per week, we assume they drank 2.5 drinks per week on average. 2. This was measured in a variety of ways. a. In the past week, how many alcoholic beverages did you have? b. Thinking about the past year, on the average how many drinks did you have each week? 3. This phenotype was left-anchored at 1 and log-transformed prior to analysis, in order to prevent  outliers from having undue leverage on analyses. | N/A |
| Alcohol intake frequency | Definitions of dietary factors are available on the UK Biobank website (accessed via the website on the right) | <https://biobank.ctsu.ox.ac.uk/crystal/field.cgi?id=1558> |
| Processed meat intake |  | <https://biobank.ctsu.ox.ac.uk/crystal/field.cgi?id=1349> |
| Poultry intake |  | <https://biobank.ctsu.ox.ac.uk/crystal/field.cgi?id=1359> |
| Beef intake |  | <https://biobank.ctsu.ox.ac.uk/crystal/field.cgi?id=1369> |
| Non-oily fish intake |  | <https://biobank.ctsu.ox.ac.uk/crystal/field.cgi?id=1339> |
| Oily fish intake |  | <https://biobank.ctsu.ox.ac.uk/crystal/field.cgi?id=1329> |
| Pork intake |  | <https://biobank.ctsu.ox.ac.uk/crystal/field.cgi?id=1389> |
| Lamb/mutton intake |  | <https://biobank.ctsu.ox.ac.uk/crystal/field.cgi?id=1379> |
| Bread intake |  | <https://biobank.ctsu.ox.ac.uk/crystal/field.cgi?id=1438> |
| Cheese intake |  | <https://biobank.ctsu.ox.ac.uk/crystal/field.cgi?id=1408> |
| Cooked vegetable intake |  | <https://biobank.ctsu.ox.ac.uk/crystal/field.cgi?id=1289> |
| Tea intake |  | <https://biobank.ctsu.ox.ac.uk/crystal/field.cgi?id=1488> |
| Fresh fruit intake |  | <https://biobank.ctsu.ox.ac.uk/crystal/field.cgi?id=1309> |
| Cereal intake |  | <https://biobank.ctsu.ox.ac.uk/crystal/field.cgi?id=1458> |
| Salad / raw vegetable intake |  | <https://biobank.ctsu.ox.ac.uk/crystal/field.cgi?id=1299> |
| Coffee intake |  | <https://biobank.ctsu.ox.ac.uk/crystal/field.cgi?id=1498> |
| Dried fruit intake |  | <https://biobank.ctsu.ox.ac.uk/crystal/field.cgi?id=1319> |
| Salt added to food |  | <https://biobank.ctsu.ox.ac.uk/crystal/field.cgi?id=1478> |
| Water intake |  | <https://biobank.ctsu.ox.ac.uk/crystal/field.cgi?id=1528> |
| Acute tubulo-interstitial nephritis | The Endpoint definition for Acute tubulo-interstitial nephritis is in the Finngen biobank. | <https://risteys.finngen.fi/endpoints/N14_PYELONEPHR> |

The data used in our study were mainly processed by the MRC Integrative Epidemiology Unit (IEU) at the University of Bristol from the UK Biobank and the FinnGen biobank in 2018/2021. The data of the UK Biobank and the FinnGen biobank may also be partially updated. Therefore, the data from their official website and the data from IEU may not be completely consistent.More information on exposure and outcomes can be obtained on the website provided in the table.
